# Suicide attempt by self-stabbing of the bladder: a case report

**DOI:** 10.1186/1752-1947-8-391

**Published:** 2014-11-27

**Authors:** Youssef Kharbach, Somuah Tenkorang, Mustapha Ahsaini, Soufiane Mellas, Jalaleddine El Ammari, Mohammed Fadl Tazi, Abdelhak Khallouk, Mohammed Jamal El Fassi, Moulay Hassan Farih

**Affiliations:** 15 Department of Urology, Hassan II University Hospital, Fez, Morocco; 16 Department of Anatomy, Faculty of Medicine, Mohammed Ben Abdellah University, Fez, Morocco

**Keywords:** Foreign body, Urinary bladder

## Abstract

**Introduction:**

The presence of foreign bodies in the bladder often falls within questionable practices in psychiatric settings or in iatrogenic instances such as during endoscopy or migration of foreign bodies around the bladder remaining after surgery on organs close to the bladder. Psychiatric disorders have been reported in patients admitted for self-introduction of foreign bodies in the bladder during an act of sexual satisfaction. However, to the best of our knowledge, no similar case in the context of suicide has been reported in the English-language literature.

**Case presentation:**

A 56-year-old Moroccan man known to have untreated paranoid schizophrenia and a history of several previous suicide attempts was presented to the emergency unit of our hospital after self-stabbing with a 15cm sewing needle. His stab wound was located at the hypogastric region of the abdomen, with full penetration of the needle into the abdomen. A computed tomographic scan showed a breach on the dome of the bladder responsible for extravasation of the contrast dye, which revealed a peritoneal cavity effusion of average abundance and a suspected lesion of the left pelvic ureter. An exploratory laparotomy was performed. Approximately 1000mL of widely dispersed fluid was observed in the abdominal cavity. During exploration of the bladder, two centimetric intrabladder breaches were found, one of which was a breach of the left pelvic ureter without other associated lesions. The breaches were sutured, and a ureteral catheter was mounted. The patient’s post-operative follow-up was unremarkable.

**Conclusions:**

The wide variety of ways that foreign bodies are introduced into the lower urinary tract pose diagnostic and therapeutic difficulties for the urologist. Management of these patients is facilitated by the use of endoscopy.

## Introduction

The discovery of foreign bodies in the bladder always raises questions about the circumstances of introduction or migration of these materials, as well as the psychological profile of the patient. This condition can be life-threatening. Timely and appropriate medical care should be accompanied by psychiatric care in such cases.

## Case presentation

**Figure 1:**
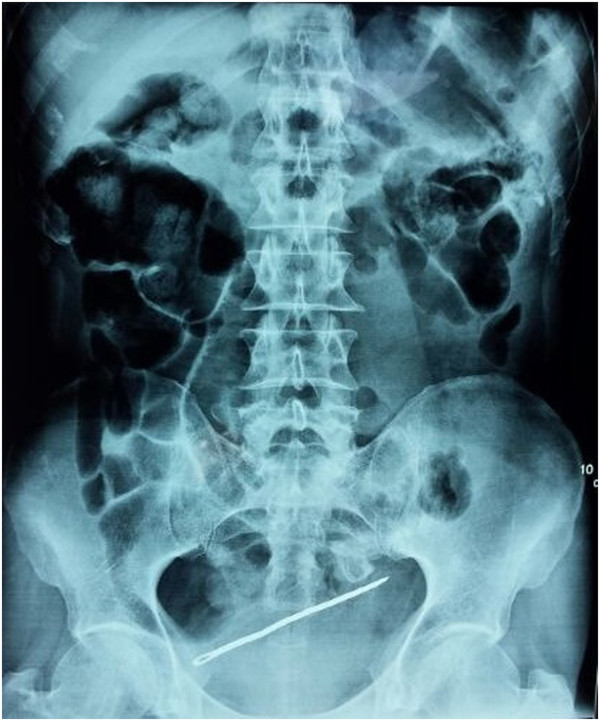
**X-ray of the patient revealing a sewing needle projecting into the bladder area.**

**Figure 2:**
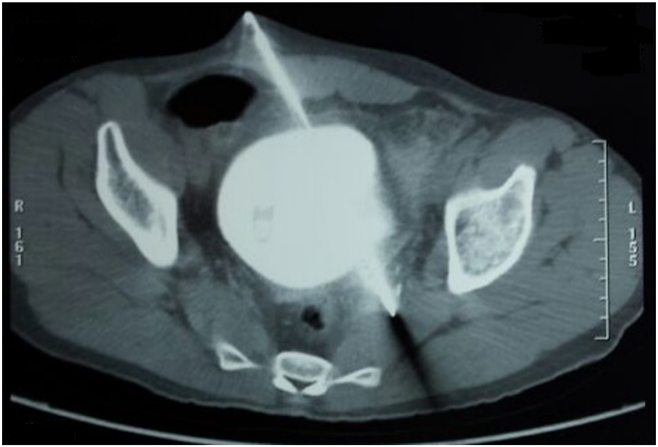
**Excretory phase computed tomographic scan showing lesions of the bladder and the left ureter.**

**Figure 3:**
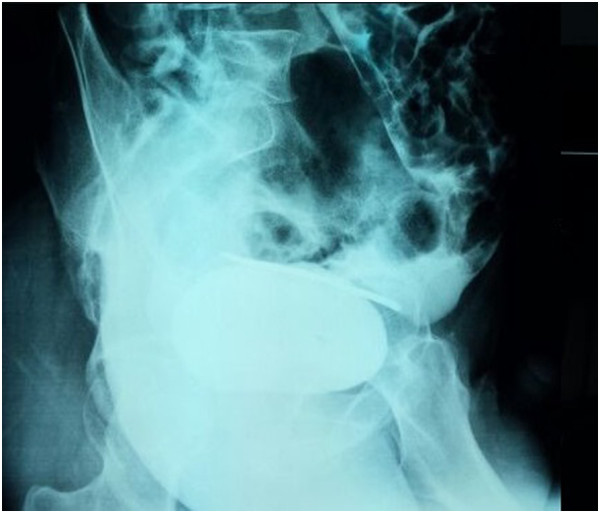
**Cystogram showing the sewing needle and contrast extravasation dye.**

A 56-year-old Moroccan man known to have untreated paranoid schizophrenia with a history of several suicide attempts was presented to the emergency room of our institution six hours after self-stabbing with a 15cm sewing needle that had entirely penetrated into his abdomen. Upon clinical examination, he was found to be conscious with incoherent speech and stable vital signs. During his abdominal examination, three entry points were found at the hypogastric region (one on the midline and two on the right side) with generalized abdominal tenderness. The patient presented neither hematuria nor rectal bleeding.An X-ray of the abdomen revealed a sewing needle projecting into the bladder area with no signs of pneumoperitoneum (Figure 1).A computed tomographic (CT) scan and a cystogram both showed a breach on the dome of the bladder visualized as extravasation of contrast dye, peritoneal effusion of average abundance and a suspected lesion of the left pelvic ureter, with no lesions in the digestive tract (Figures 2 and 3).

The patient was admitted to the operating room, where an exploratory laparotomy was performed. Approximately 1000mL of widely dispersed fluid was observed in the abdominal cavity. During the exploration of the bladder, two centimetric intrabladder breaches and a breach on the left pelvic ureter were found without other associated lesions. These breaches were repaired by surgical suture, and a ureteral catheter was mounted. The incision was closed primarily in two layers, and a urinary catheter was placed. No problems were encountered during the patient’s peri-operative clinical course. The patient received psychiatric care, and his post-operative follow-up was unremarkable.

## Discussion

Foreign bodies in the bladder can occur either by self-introduction or by migration from adjacent organs [1]. Introduction of foreign bodies can be voluntary and related to a psychiatric disorder or can occur by accidental penetration of objects or iatrogenic trauma due to firearms [2]. This presentation can occur in various situations and can sometimes be life-threatening [3]. It often raises questions about the psychological profile of the patient. Psychiatric disorders have been reported in patients admitted for self-introduction of foreign bodies into the bladder as an act of sexual satisfaction [4]. However, to the best of our knowledge, no case involving a patient with suicidal intent has been reported in the English-language literature to date. Bladder rupture may be intraperitoneal, extraperitoneal or mixed intra- and extraperitoneal [5].

The clinical presentation of these injuries may vary from simple urinary symptoms suggestive of cystitis to signs of acute abdomen [3]. Signs of bladder injuries may be overlooked, especially in the context of polytraumatized patients, but the presence of the triad of macroscopic hematuria, suprapubic pain and voiding dysfunction suggest the diagnosis and indicate exploration of the bladder [5].

X-rays show a radio-opaque foreign body [2], but they may be unremarkable when the foreign body is radiotransparent. In this situation, an ultrasonogram can objectify bright echogenic foci with distal acoustic shadowing [2].

CT scans of the bladder can be taken in two ways, either by intravenous contrast injection or retrograde filling by contrast dye. The intravenous route is very imprecise in showing bladder rupture because bladder distension is insufficient. A cystogram is preferable in this situation [5] because it has a sensitivity of 95% and a specificity of 100% [6].

The intra- or extra-peritoneal nature of the injury, which is easily visualized on the cystogram, determines the therapeutic approach to be used. A pelvic CT scan provides valuable information on the status of other pelvic organs as well as the pelvis itself [5]. Cystoscopy may also help in making the diagnosis in the context of radiotransparent foreign bodies by showing their nature and size, and it may also aid in identifying complications. It can be performed for therapeutic objectives for extraction of these bodies when no associated lesions are found, and it may be used in cases of extra-peritoneal bladder breach [7].

The therapeutic approach depends on the patient’s condition, the assessment of the lesions, and the size, shape and nature of the intravesical foreign body [8]. The extraction of a foreign body in the bladder can be performed through surgical exploration. A suprapubic cystostomy may be performed for large foreign bodies, when one of the extremities of the bodies is engaged in the bladder wall and when attempts at endoscopic extraction fail [9].

Finally, it is necessary to treat any existing urinary tract infection by using appropriate antibiotics as well as tetanus vaccination, and psychiatric care should be requested to prevent a recurrence when the injury is a result of a voluntary act [10].

## Conclusions

The wide variety of ways that foreign bodies are introduced into the lower urinary tract always raise curiosity because such injuries are often related to sexual or erotic acts in a psychiatric context. To the best of our knowledge, no previous case of attempted suicide by self-stabbing with foreign bodies has been reported to date. Radiology and endoscopy facilitate the diagnosis and therapeutic care in these patients.

## Consent

Written informed consent was obtained from the patient’s legal guardians for publication of this case report and any accompanying images. A copy of the written consent is available for review by the Editor-in-Chief of this journal.

## Abbreviations

CT: computed tomography.
